# Psychoanalysis and Affective Neuroscience. The Motivational/Emotional System of Aggression in Human Relations

**DOI:** 10.3389/fpsyg.2018.02475

**Published:** 2019-01-14

**Authors:** Teodosio Giacolini, Ugo Sabatello

**Affiliations:** Department of Human Neuroscience, Sapienza University of Rome, Rome, Italy

**Keywords:** adolescence, aggression, motivational system, dominance, submission

## Abstract

This article highlights the evolutionary biological epistemology in Freud psychoanalytic theory. The concepts of aggressive and sexual drives are cornerstones of the psychoanalytic epistemological system, concerning the motivational/emotional roots of mental functioning. These biological roots of mental functioning, especially with regard to aggressive drive, have gradually faded away from psychoanalytic epistemology, as we show in this article. Currently, however, Neurosciences, and in particular Affective Neuroscience (Panksepp, 1998), can help us to have a better understanding of the biological roots of human mental functioning. The motivational/emotional systems studied by Affective Neuroscience can give a new epistemological foundation to the aggressive drive concept in psychoanalytic theory. Over the course of human evolution, motivational/emotional systems have played a role in social relationships and also in mental functioning. In this regard, among the various types of aggression (ANGER in Panksepp taxonomy 1998) that we consider in our article, *inter-male* aggression, also named *Dominance motivational/emotional system*, is that which regulates social interactions between sexually matured adults. This type of aggression acts in complementary connection with FEAR motivational/emotional system that regulate submissive behavior and social defeat, and the latter one is of the more important stressors. The interaction between aggression and FEAR motivational/emotional systems gives rise to agonistic behavior or dominance/submission motivational/emotional system, as we propose in our article. There is now a large literature that identifies in the dynamic of *Competitive behavior*, which is one of the main factors of mental illness. When social interactions activate the competitive behavior, the subject can perceive himself as “destined to victory” or “destined to defeat,” activating either behaviors or emotions connected to the *Involuntary Defeat Strategy* or *Involuntary Dominant Strategy* (Sloman, 2002), which we can find in many types of mental disorders, for example, mood disorders or anxiety disorders.

## Introduction

In the history of psychoanalysis, *aggression* has progressively acquired a central importance. The psychodynamics of *libido* dominated and characterized the very identity of the psychoanalytic movement from the beginning (Freud, 1895, 1905). Very soon after the initial study of *libido, aggression* (Freud, 1920) became the ineradicable complement, an element of mental functioning fundamental to understand both normal and pathological functioning. Between the two concepts, however, there was a profound difference from the beginning. Libido has been a concept that has derived its heuristic strength from having its biological base in sexuality. Aggressiveness, on the other hand, has implicated in psychoanalytic theory an initial tendency to trace back into the biological sciences the roots of its presence in the human species, as will be highlighted in the present work, but only then to be clearly eradicated in favor of an option not shared by the biological sciences and which has increased the psychological vertex of psychoanalysis. The trend of the theorization of *aggression* in Freud proposes, in some respects, the parable of the theory of normal and pathological mental functioning in his thought, first based on a biological assumption, the instinctual and neurological one (Freud, 1895), subsequently largely abdicated and replaced by the psychological *structural model* (Freud, 1923). The latter was affirmed on the assumption, increasingly supported by Freud after the famous assertion of inability to pursue a neurological vertex (1895) in the study of unconscious dimension of the mind, and the only way it could be explored would be through the elaboration of psychological theories, that are definable and verifiable through clinical work. This methodological position of Freud, understandable and shareable for the historical period in which he operated, has become a *dogma* for the psychoanalytic community. Hence the difficulties that psychoanalytic theory currently has in receiving and using neuroscientific discoveries, which mainly reside in the lack of *epistemology*, thus preventing the link of psychopathological and clinical theories with the new discoveries of basic research.

An important contribution to the construction of this *epistemological ring* was given by the work of Bowlby (1969). Bowlby, as he is known, wanted to refound the psychoanalytic theory starting from the instincts from which Freud had started and from Darwin’s lesson that is the *comparative psychology*. It should be remembered, as already indicated by Bowlby (1969), that in the second half of the twentieth century, disciplines were established that did not exist at the time of Freud, that is, ethology and neuroethology (from which derives *Affective Neuroscience*; Panksepp, 1998). These disciplines share an element that can be traced even in the foundations of psychoanalysis, the rooting in the evolutionary format. The evolutionary epistemology (Campbell, 1975; Lorenz, 1977) has demonstrated, especially now with the rapid development of neuroscience, to have an exceptional heuristic power, able to provide that link between the *BrainMind* (Panksepp, 1998) physiological functioning and the psychopathology.

### Freud and Evolution

Freud began the study of psychopathology as a convinced environmentalist, believing that the external environment provoked mental disturbances, especially *via* sexual experiences (see Freud’s seduction theory). This theory coincided with similar theories stated by other psychiatrists and sexologists of the same era (Von Krafft-Ebing, Binet, Havelock Ellis, Moll, etc.) that suggested that sexuality was the principle cause of mental disorders (Sulloway, 1979). Why the sexual experiences were considered potential origin of mental disorders? The sexual instinct, considered responsible for psychopathologies, was a theory derived by Darwin during the late nineteenth century. The great English biologist had identified and brought to the attention of the scientific world two factors that regulate the relational life of the animals and also of the human species: *natural selection* (Darwin, 1859) based on the *instinct of self-preservation* and *sexual selection* (Darwin, 1871). In the dynamic between these two instincts, post-Darwinian psychiatry identified the genesis of the various forms of psychopathology. Toward the end of the nineteenth century, however, the interest in the study of sexual instinct became more and more prevalent. Hence, the development of sexology and the parallel tendency is to see the primary cause of mental distress in the sexual instinct. The explanation of this point of view is attributable, at least in part, to the work of Darwin (Sulloway, 1979), who had argued that the reproductive system was that particularly subject to variation: “Several reasons make me believe in this; but the chief one is the remarkable effect which confinement or cultivation has on the functions of the reproductive system; nothing is more easy than to tame an animal, and few things more difficult than to get it to breed freely under confinement, even in the many cases when the male and female unite” (Darwin, 1859, p. 8).

Freud was profoundly influenced by the Darwinian and post-Darwinian culture of his time. We summarize some dates and events. Freud was born in 1856. In 1859, Darwin published the *Origin of the species*; in 1871, *Origin of man*; and in 1872, *The expression of emotions in humans and animals*. In 1873, Freud enrolled in the medical faculty of the University of Vienna and the first voluntary course he followed was entitled “General Biology and Darwinism” (Sulloway, 1979). The interest in Darwinian biology prompted Freud’s desire, at the beginning of his scientific training, to become a research biologist (Jones, 1953; Anzieu, 1976). Freud spent many years in the laboratory of Professor Ernst Brücke (1819–1892), biologist and physiologist, with the hope of becoming a researcher at his university chair, but unfortunately came to no avail. During those years, Freud carried out some important research on the neuroanatomy of the *ammocoete (Petromyzon planeri)* larval form of stream lamprey, on the structure of the eel gonads, and a study of the *crayfish* nerve cells, as well as the processing of cell staining methods for histological research. Freud devoted a lot of time and study to *comparative biology* subsequently failing to reach a professional level in medicine. Hence, his orientation is toward the more limited field of neurology and psychiatry. Psychoanalysis was born, therefore, “from a failed biologist” (Sulloway, 1979). But it was during this psychoanalytic prehistory of Freud’s intellectual life that the neurobiological and evolutionary format of his psychotherapeutic creature took root, roots that can be powerfully revived by current neuroscience.

Darwin’s influence on Freud’s thoughts is particularly visible in his dual instinctual theory. In fact, Freud elaborated a motivational theory of dual-type drives, analogous to that of the English biologist. This drive dualism would be present transversely throughout his work.

Freud very soon opposed the *sexual libido* (which pushes the subject to sexual satisfaction even at the risk of one’s own personal protection) to *the instincts of self-preservation*. With this last term, Freud designated the set of needs linked to the somatic functions necessary for the individual preservation of the life. The prototype of such drives is hunger. In *The Psycho-Analytic View of Psychogenic Disturbance of Vision* (Freud, 1910), Freud speaks briefly, for the first time, of both *sexual drive* and *the instincts of self-preservation*. Freud in his work *Three Essays on Sexuality* (Freud, 1905) had already pointed out how: “…sexual instincts. At their first appearance they are attached to the instincts of self-preservation…” (Freud, 1915a, p. 125), like the feeding instinct. Freud has never extensively discussed *the instincts of self-preservation* (see Laplanche and Pontalis, 1967), merely referring to hunger and other great somatic functions such as defecation, urination, muscular activity, etc. In 1911, Freud in *Formulations on the Two Principles of Mental Functioning* states that *the instincts of self-preservation* can be satisfied only with a real object, making a rapid transition from the *Pleasure Principle* to *Reality Principle*, while the sexual drives can continue to be satisfied also by fantasy (Freud, 1911, p. 222). One aspect of *the instincts of self-preservation*, connected to the one described above, is characterized by *fixed objects* and *instinctual aims* (see “fixed action patterns” Tinbergen, 1951; Lorenz, 1981), and this feature brings them closer to the concept of *Instinkt* than to that of *Trieb* (drive). Freud quotes “If inherited mental formations exist in the human being—something analogous to instinct in animals—these constitute the nucleus of the Ucs*”* (Freud 1915b, p. 194). (In German tradition, Tribe is the term for naming instinct in humans, whereas Instinkt is reserved to nominate instinct in animals). On the contrary, for Freud, the sexual drives (*Trieb*) are characterized by a relative indeterminate pressure that can be expressed in different ways, and its aim can be achieved through different actions. *The self-preservation instincts* for maintaining the physical integrity of the individual were named *Ego instincts* (Freud, 1910) by Freud, who emphasized their being in conflict with sexual drives. Freud quotes “From the point of view of our attempted explanation, a quite especially important part is played by the undeniable opposition between the instincts which subserve sexuality, the attainment of sexual pleasure, and those other instincts, which have as their aim the self-preservation of the individual—the ego-instincts. As the poet has said, all the organic instincts that operate in our mind may be classified as ‘hunger’ or ‘love’” (Freud, 1910, pp. 213–214). With the conflict between ego and sexuality, Freud reiterated the Darwinian dialectic between natural selection, which is concerned with the preservation of the fittest individual, and the sexual one, which aims to conserve the species: “The individual does actually carry on a twofold existence: one to serve his own purposes and the other as a link in a chain, which he serves against his will, or at least involuntarily… The separation of the sexual instincts from the ego-instincts would simply reflect this twofold function of the individual” (Freud, 1914, p. 78).

And, in another famous passage, Freud points out to be deeply permeated by Darwinian ideas, he states *“*I should like at this point expressly to admit that the hypothesis of separate ego-instincts and sexual instincts (that is to say, the libido theory) rests scarcely at all upon a psychological basis, but derives its principal support from biology” (Freud, 1914, p. 79).

In the years between 1910 and 1915, Freud completed his *First Drive Theory*, and during this period, he wrote an important book *Totem and Taboo* (Freud, 1912–13). In this work, and also in latter books such as *The Future of an Illusion* (Freud, 1927) and *Moses and Monotheism* (Freud, 1939), Freud showed his interest in the *evolutionary-anthropological thought* (Sulloway, 1979) and for the possibilities that it provides to be able to reconstruct the roots of human mental operations. In *Totem and Taboo*, Freud takes up the hypothesis formulated by Darwin in *The origin of man* (Darwin, 1871), on the beginnings of *homination*, in which the human species must have lived in hordes, led by a strong and powerful male, who controlled access to females of younger and subordinate males. The young and subordinate males were forced to move away from the group in order to copulate and consequently form new hordes. It was hypothesized that the birth of exogamy, which practically characterizes most cultures, was a derivative of these interactive dynamics. Freud made this reconstruction his own, which he enriched with the hypothesis of parricide by the group of young allied males, from which the birth of the totemic religion as a remedial act of the primordial event. *Totem and taboo*, therefore, highlights in an extremely clear way, Freud’s effort to build clinical psychoanalysis on the foundation of an *evolutionary-anthropological thought*, enabling Freud to answer crucial questions on both clinical work and the development of psychoanalytic theory. Previous attempts to respond to these problems through a purely neurophysiological explanation precociously failed, as evidenced by the *Project for a Scientific Psychology* (Freud, 1950 [1895]). From that moment on, Freud sought answers to these and other problems, no longer in a neurophysiological reductionism, impossible to practice in his time, but in a historical-evolutionary model (Sulloway, 1979). Freud writes about the question on the causes of neurosis: “I am prepared with an answer which I know will seem daring to you. I believe these primal phantasies, as I should like to call them, and no doubt a few others as well, are a phylogenetic endowment. In them the individual reaches beyond his own experience into primaeval experience at points where his own experience has been too rudimentary. It seems to me quite possible that all the things that are told to us today in analysis as phantasy—the seduction of children, the inflaming of sexual excitement by observing parental intercourse, the threat of castration (or rather castration itself)—were once real occurrences in the primaeval times of the human family, and that children in their phantasies are simply filling in the gaps in individual truth with prehistoric truth. I have repeatedly been led to suspect that the psychology of the neuroses has stored up in it more of the antiquities of human development than any other source” (Freud, 1916, pp. 370–371). This model was strongly impregnated, as we see, from the *Second Law of Inheritance of acquired characteristics*, the pre-Darwinian evolutionary theory of Lamarck’s 1744–1829), that is, the repeated historical experiences are deposited in the hereditary patrimony of the species. Thus, Freud identified the roots of the current *Oedipus complex*, at the base of man’s neurosis, in distant human prehistory. Freud wrote about it, also identifying the roots of the mechanism of *repression*: “the children have entered upon a new phase of development in which they are compelled to recapitulate from the history of mankind the repression of an incestuous object-choice, just as at an earlier stage they were obliged to affect an object-choice of that very sort” (Freud, 1919, p. 108).

In *Totem and Taboo* (Freud, 1912–13), Freud does not discuss the problem of *aggression*, but nevertheless he gives an extremely significant evolutionary representation of it. The *story* begins with a dyadic comparison between two males in which the defeated (the young adolescent son) is forced to leave the territory in favor of the stronger one, the father, leader of the group, which we could define as territorial aggressiveness (Archer, 2009). Later on, the *story* tells of “the brothers” allying against the leader, that is, Freud described a new type of competition in which the *aggression* is modulated by the capacity of the contenders in order to create alliances against a common enemy (Flinn et al., 2012). Only after *Totem and Taboo* (Freud, 1912–13), Freud would come to define the problem of *aggression* as explicitly relevant for the self-preservation instincts in *Instincts and their vicissitudes* (Freud, 1915a).

### Freud and Aggression

Until 1915, Freud had theorized about *aggression* by linking it to sexuality. Freud was interested in aggressiveness that he was finding in the study of sexual perversions, a topic of extreme relevance in psychiatry of the late nineteenth century, with the famous terms *sadism* and *masochism* coined by the psychiatrist and sexologist R. von Krafft-Ebing (1840–1902). Freud writes “1 [The last two sentences were given their present form in 1915. In 1905 and 1910 they read as follows: *‘*It may be assumed that the impulses of cruelty arise from sources which are in fact independent of sexuality, but may become united with it at an early stage owing to an anastomosis [cross-connection] near their points of origin. Observation teaches us, however, that sexual development and the development of the instinct of scopophilia and cruelty are subject to mutual influences which limit this presumed independence of the two sets of instincts.’]” (Freud, 1905, p. 193). And about the sadism Freud writes “As regards active algolagnia, sadism, the roots are easy to detect in the normal. The sexuality of most male human beings contains an element of aggressiveness—a desire to subjugate; the biological significance of it seems to lie in the need for overcoming the resistance of the sexual object by means other than the process of wooing. Thus sadism would correspond to an aggressive component of the sexual instinct which has become independent and exaggerated and, by displacement, has usurped the leading position” (Freud, 1905, pp. 157–158).

Only in 1913, Freud returned to the problem of *aggression* in *The Disposition to Obsessional Neurosis, a Contribution to the Problem of the Choice of Neurosis* (Freud, 1913), where he introduces the concept of *pre-genital organization*. In it, Freud, speaking of the phase of sexual development in which the subject, who develops later an obsessional neurosis, is fixated, identifies this libidinal stage in the *sadistic-anal organization*. At this stage of the sexual libido development, it is not yet possible to talk about female or male object choice because we are still at a level of pre-genital organization, so it is only possible to talk about trends with an active goal and trends with a passive goal, which will go subsequently to characterize the male and female gender identity. Freud quotes: “Activity is supplied by the common instinct of mastery, which we call sadism when we find it in the service of the sexual function; and even in fully developed normal sexual life it has important subsidiary services to perform. The passive trend is fed by anal erotism, whose erotogenic zone corresponds to the old, undifferentiated cloaca” (Freud, 1913, p. 322). Until then Freud had dealt with the theme of *aggression*, linking it primarily to its function in relation to the sexual drive, which determines its sadistic and secondarily masochistic modalities.

In 1915, Freud in *Instincts and their Vicissitudes* came to the important conclusion about the belonging of the *aggression* to *the instincts of self-preservation* or *Ego instincts*: “Indeed, it may be asserted that the true prototypes of the relation of hate are derived not from sexual life, but from the ego’s struggle to preserve and maintain itself” (Freud, 1915a, p. 137). *Aggression* considered as an *instinct of self-preservation* or *Ego instincts* is a conceptual position that completely keeps up with a Darwinian biology viewpoint. But at the same time, Freud describes what he means by hatred, being an effect of unpleasant sensations that are perceived by the ego due to the presence of the external reality objects. During these times, he completely developed the theorization of the first phases of libidinal development through the conceptualization of *narcissism* (Freud, 1914). *Narcissism* is formed at the beginning by pleasurable sensations and followed by identifying gratifying objects of external reality. Freud quotes “Hate, as a relation to objects, is older than love. It derives from the narcissistic ego’s primordial repudiation of the external world with its outpouring of stimuli. As an expression of the reaction of unpleasure evoked by objects, it always remains in an intimate relation with the self-preservative instincts; so that sexual and ego-instincts can readily develop an antithesis which repeats that of love and hate. When the ego-instincts dominate the sexual function, as is the case at the stage of the sadistic-anal organization, they impart the qualities of hate to the instinctual aim as well” (Freud, 1914, p. 139). *Aggression* is thus conceptualized as a drive of primary and biological self-preservation, functional to survival for the human organism. At the same time, *aggression* is equated with the effect of hate, a consequence of the external world objects that are a source of perturbation stimuli for the subject. Here, we notice Freud’s difficulty in combining a biological-evolutionary vertex with the psychological-speculative one.

In the following years, Freud revised the theory of *drives, second drive theory* being mentioned in the book *Beyond the Pleasure Principle* (Freud, 1920). In this work, Freud shifts *aggression* from the drives of self-preservation to a new drive organization that can be identified with the *Death instincts*. The *instincts of self-preservation* or *Ego instincts*, instead, converge together with the sexual drives in a new great grouping called *Life drives*. Without wishing to enter into a work as controversial as *Beyond the Pleasure Principle* (Freud, 1920), it needs to be mentioned that it was the logical consequence of H. Haeckel’s fundamental *Biogenetic law* for which ontogenesis recapitulates phylogeny. Freud pushed these assumptions of the biogenetic theory to the extreme, inferring that if an organism in its development is forced to recross phases of a distant evolution, this implies that an aspect inherent in organic life itself and of the drive in particular is that of restoring the previous state of things. Freud quotes “It seems, then, that an instinct is an urge inherent in organic life to restore an earlier state of things which the living entity has been obliged to abandon under the pressure of external disturbing forces; that is, it is a kind of organic elasticity, or, to put it another way, the expression of the inertia inherent in organic life” (Freud, 1920, p. 36). In this new theory, the sexual drives no longer conflicted with *the instincts of self-preservation*, but both are part of the *Life drives* except, however, *aggression*. It was removed from *the instincts of self-preservation* and inserted into the *Death instincts,* but with a peculiarity. Only after the combination of *Death instincts* with libidinal ones aimed at the outside world, the disintegrating tendency inherent in the *Death instincts* becomes *aggression* turned toward the objects of the external world. The *Second drive theory* and the concept of the *Death instincts*, therefore, show how Freud thought, while affirming the primacy of the psychological dimension for psychoanalysis, concealing intellectual binary tracks constituted by biological interests and constructs, such as that represented by the *biogenetic law* (Sulloway, 1979). The problem was that these biological assumptions at the base of these last works of Freud not only constitute a kind of cryptobiology (Sulloway, 1979), but also above all they were not shared by the biological sciences contemporary to him.

### Aggression in Post-Freudians

The problem of *aggression* has become, as considered above, ever more important in psychoanalytic thinking, as more and more complex mental operations were described, and so more problematic psychopathologies were treated. Let us remember only how the work of Melanie Klein (1882–1960) marked an era close to Freud’s death, providing, in the wake of the conceptualization of the *Death instincts,* a theory of *aggression* that placed this drive in a central position in the developmental dynamics of the human mind (Klein, 1932). Klein identified, in the preoedipal and preverbal stages of development, the central moments for the future functioning of the mind, that is the developmental phases in which the importance of the dynamics of the aggressive drive is paramount. This Kleinian and post-Kleinian revolution in considering the aggressive drive from a developmental point of view has allowed, at the same time, a developmental conceptualization of the psychotic states of the mind connected to these very early stages of mental development and therefore the possibility of elaborating hypotheses of cure. *Aggression* has, therefore, been identified as a drive with its developmental and interactive dynamic, implicitly recognizing it as being the manifestation of a biological substrate, without, however, following it with an interest in deepening the knowledge that the natural sciences were supplying about it. One of the consequences of this conceptualization was the progressive sclerosis of the developmental hypothesis, which led to a rigid pathogenic ideology that saw in the relational events of early childhood the causes of mental disorders, looking for the causes of serious discomfort in a problem of *aggression* placed in a mythical initial period of mental life. Currently, this approach is combined with researches that highlight the peculiar contribution of the later developmental moments (see, e.g., Fonagy, 2005, p. 214) and the importance of current interpersonal relationships (Lewis, 1998).

In recent decades, research on the etiopathogenesis of psychopathology has directed its attention to the motivational system of *attachment*, which has shown a high correlation between insecure and disorganized attachments and subsequent mental disorders, in particular, personality disorders (Fonagy, 2001). The massive effort given to research on attachment in recent decades has allowed psychoanalysis to benefit from basic empirical research, but has simultaneously overshadowed the problem of *aggression.* The latter has become a secondary problem regarding the regulation of emotions, which is implicitly considered to be a function of the dynamics of attachment. As a consequence, a sort of theoretical vacuum has been created, in which the blanket of attachment theory has been extended to explain the onset of psychopathology (Cowan and Cowan, 2007), especially in adolescence.

The discovery of the attachment motivational system has shown that the human species, similar to the other mammalian species, are born with biologically innate system that regulates the interactions between parents and offspring. But, there is another biologically innate system, whose function is to regulate interactions among the adults in the mammalian groups, including human species, called *Inter-male aggression* or *Agonistic behavior* (Scott and Fredericson, 1951). This type of *aggression* becomes mature with puberty and replaces the attachment system in the primacy of the regulation of the emotional states in the subject (Stevens and Price 1996), even if the attachment system will stay in function for all the life, for example, in the romantic love or in the adult health difficult moments. The research area that substantiates this theory is, as we will see, widely interdisciplinary and allows us to reframe the broad observations of clinical psychoanalysis on psychopathology with basic research data.

### Aggression as a Regulator of Relationships

Briefly we will outline the theoretical scientific area of reference for a deepening of the problem of *aggression*. Evolutionism, identifying the possibility of reconstructing generative lines among different animal species including the human ones (LeDoux, 1996), is responsible for the comparative study of somatic, neurophysiological, behavioral, and emotional functioning (Lorenz, 1963; Panksepp and Biven, 2012). A neurotologist that contributed to a significant increase in the behavior comparative studies was Paul MacLean (MacLean, 1990a,b), universally known for having unified what he himself called in 1952 the *limbic system* (Roxo et al., 2011), the heart of the mammal’s and human’s emotional system. Paul MacLean developed an evolutionary model of the human nervous system of great heuristic significance which was greatly but not completely shared by the scientific community (Cory, 2002; Panksepp and Northoff, 2009). he identified three brain areas from an anatomical, functional, and phylogenetical perspective, that are connected with evolutionarily related species. The oldest phylogenetical part of the *triune brain* is located in the deepest part of the human brain called the *striatal system* and the *brainstem*, that MacLean calls *Complex R* or *reptilian brain* (MacLean, 1990a,b). Reptiles are characterized by the prevalence of this neuroanatomical structure, which show typical behaviors related to intraspecific communication: *territorial marking, challenge behavior, courtship, and submission behavior*. MacLean identifies these behaviors as *Paleomentation* (MacLean, 1990a,b, p. 12) to indicate the origins of mental and communicative activity. In fact, the four types of behavior have their meaning only among individuals of the same species, including human species, as we will consider. MacLean identifies, therefore, in the phylogenetically oldest part of the brain, the *R complex*, the oldest structures of the regulation of intra-species *aggression*, which is the *territorial agonism*, from which the agonism for the formation of rank hierarchies will subsequently evolve.

Over the neuronal organization of the *reptilian brain*, a structure called the *limbic system* was evolving and characterized by the presence of *cingulum gyrus, amygdala, hippocampus, parts of the hypothalamus, area of the septum, and nucleus accumbens (part of the striatum).* The evolution of the limbic system (MacLean, 1985, 1990a,b) allows the offspring to recognize their caregiver. In this way, the individualized recognition of the conspecific (Gherardi et al., 2010) has become an acquisition of mammals and bird species, evolution at the base of the ability to form social networks in which the individual recognizes the other and knows he is recognized by the conspecific. This individual recognition is central, as we will see below, for the formation of dominance hierarchies that structures the group. The *limbic system* characterizes the passing from a herd formed by anonymous individuals, like fish, to mammals in which each social network involves the memory in each participant of the hierarchy in which he/she is inserted.

*The R-complex* and the *limbic system* form what is currently called *subcortical-cortical midline structures* (SCMS) (Northoff and Panksepp, 2008; Panksepp and Northoff, 2009; Alcaro et al., 2017), homologous structure in mammals and in human species, and considered “the foundation for epigenetic emergence… of selfhood across different individuals within a species” (Panksepp and Northoff, 2009, p. 193).

Finally, a third organization has been increasingly evolving in the course of phylogenesis, consisting of the *neocortex* (MacLean, 1990a,b), of which particular interest is the *cortical midline structures* (CMS) that have intimate functional relationships with the lower structures of the *subcortical-cortical midline structures* (SCMS) (Northoff and Panksepp, 2008; Panksepp and Northoff, 2009) in the development of the selfhood.

### Motivational/Emotional Systems

Affective Neuroscience, a term coined by Panksepp (1998), the ideal continuator of MacLean’s work (Panksepp and Northoff, 2009), has in these last twenty years highlighted the presence of multiple motivational/emotional systems which, not only regulate the adaptation of species—including human species—to the environment, but also to the social environment (Liotti and Gilbert, 2011). There is now the knowledge of the neuronal circuits, the neuromediators, and the hormone components involved in these systems (Panksepp, 1998). The motivational/emotional systems identified and on which there is scientific consensus are SEEKING (capitalization is a convention established by Panksepp for labeling neurologically based emotional primes), LUST, RAGE, FEAR, maternal CARE, separation-distress PANIC/GRIEF, and physical PLAY (Panksepp, 1998; Solms and Turnbull, 2002; Panksepp and Biven, 2012). These systems can be divided into two large groups. Those with positive emotional value/reward, that is, SEEKING, LUST, maternal CARE, and physical PLAY, characterized by the release of endogenous opioids and activation of the dopaminergic system. Those with negative emotional valence/punishment, that is, RAGE, FEAR, and separation-distress PANIC/GRIEF, characterized by the activation of the stress axis *Hypothalamus-Pituitary-Adrenal Gland.* The motivational/emotional systems described above are believed to be the organizational structure of the human personality (Davis and Panksepp, 2018), and for this reason, a psychological test was carried out for the evaluation of individual personality differences, based on the research indicated above, called *ANPS-Affective Neuroscience Personality Scales* (Davis et al., 2003; Davis and, Panksepp, 2011) validated for the Italian population for both adolescence and adult age (Giacolini et al., 2017).

Alongside the systems described above, there is an eighth system, deriving from the integration of the RAGE and the SEEKING systems and therefore considered nonprimary, called the *Dominance motivational system* (Malatynska et al., 2005; Johnson et al., 2012; Van der Westhuizen and Solms, 2015). The *Dominance* system is in complementary interaction with the *Submissive behavior* (Allan and Gilbert, 1997; Malatynska et al., 2005; De Boer et al., 2016), which is a manifestation of the FEAR emotional system, (Panksepp, 1998, p. 208). Panksepp quotes “It makes good evolutionary sense for FEAR and RAGE circuit to be intimately related, for one of the functions of anger is to provoke fear in competitors, and one of the functions of fear is to reduce the impact of angry behaviors from threatening opponent” (Panksepp, 1998, p. 208). Then, we can speak synthetically of *Dominance and Submissive motivational system*, which regard the same behaviors and has the same functions of *Inter-male aggression* or *Agonistic behavior* (Scott and Fredericson, 1951), and which have the function of regulating access to food and sexual resources among sexually mature conspecifics, including human species. This system matures with the sexual development of puberty, which in particular determines the proliferation of testosterone receptors in the section that goes from *the medial amygdala* to the *lateral hypothalamus* (Derntl et al., 2009).

So, the *Dominance and Submissive system* is characterized by the complementarity of two systems. The *Dominance behaviors* depends on the RAGE system (hence implicated testosterone) and also the SEEKING system (therefore implicated the dopaminergic system; Panksepp, 1998), whereas the *Submissive behavior* depends on the FEAR system (including the stress axis and corticosteroids). The *Submissive behaviors* and emotions are described as Social defeat, which generate persistent stress (Tidey and Miczek, 1997).

### RAGE, Aggression, and FEAR

The RAGE system is formed by three types of aggressiveness (Panksepp, 1998; Panksepp and Zellner, 2004): the *predatory aggression*, the *defensive rage*, and finally the *inter-male aggression*.

*Predatory aggression* does not imply activation of the *autonomic nervous system*, and in fact, it is called a *quiet-biting attack*. This brain system is confluent with another prime emotional system, the mesolimbic dopamine circuit the SEEKING system of Panksepp (1998), which runs from the *ventral tegmental area* through *the lateral hypothalamus* to *the nucleus accumbens* and other *forebrain zones*. It is interesting what neurobiology discovered about *predatory aggression*, namely that the stimulation of the center of the system that triggers the behavior of predation in felines, the *dorsolateral hypothalamus*, which activates in a noncarnivorous species looking for food or foraging (Panksepp, 1998; Panksepp and Zellner, 2004; Siegel and Victoroff, 2009). Predatory/assertive aggression is subjectively pleasurable (elicit high levels of self-stimulation in animal experiments), and in humans, it could be seen not only in the hunting behavior but also in some pathological behaviors like serial killers, in which there is a positive feeling and a lack of autonomic activation in violence against a victim.

Of particular interest is *defensive rage,* the second type of *aggression* of RAGE system. *Defensive rage,* in contrast to *predatory aggression*, is characterized by a massive activation of the autonomic nervous system, whose effect is the rapid increase in energy availability to allow a rapid strengthening of muscle energy. The defensive aggressiveness is, in fact, activated by situations in which the individual suffers a strong coercion by external agents, but also powerful frustrations of their own motivational purpose (Panksepp, 1998; Panksepp and Zellner, 2004; Siegel and Victoroff, 2009).

The third type o*f aggression* of RAGE system is *inter-male aggression* and is aimed at comparing two contenders in order to establish a priority of access to both food and sexual resources (Bjorkqvist, 2001; Rohde, 2001; Allen and Badcock, 2003). The neural pathways involved in this type of *aggressio*n run from *medial amygdala*, through the *preoptic, anterior hypothalamus area*, until the *periaqueductal gray (PAG*; Panksepp, 1998; Panksepp and Biven, 2012). The arousability of this system is controlled by *testosterone* receptors, of which this circuit is very rich.

The intra-species aggression system works in a complementary way with the FEAR motivational/emotional system. The two systems are functionally connected and form *Dominance* (Van der Westhuizen and Solms, 2015) and *Submissive motivational system* (Stevens and Price, 1996; McGuire and Troisi, 1998; Gilbert, 2000) or *Agonistic behavior* (Scott and Fredericson, 1951; Manning and Dawkins, 2012). Let us consider the FEAR system, this along with the RAGE system, which is responsible for the defense and physical integrity of the individual (Panksepp, 1998; Panksepp and Biven, 2012). Unlike the defensive RAGE system, the FEAR system can manifest itself through two behaviors. The first is activated when the object is far away and avoidable, and in this situation, the animal shows an escape behavior. The other behavior manifests itself when the frightening stimulus is not avoidable and is characterized by immobility, by freezing. The FEAR system is the expression of a neuronal circuit composed of the *lateral and central nuclei of the amygdala* and *the anterior and medial hypothalamus* down to the *periaqueductal gray* (LeDoux, 1996; Panksepp, 1998; Weisfeld, 2002).

The two ancient systems of FEAR and defensive RAGE are closely interconnected at the level of the hypothalamus and the periaqueductal gray, interconnectedness that is maintained at the level of the *amygdala* (Panksepp and Biven, 2012), where they give rise to well-organized distinct circuits (see below). The two circuits thus become functionally interconnected in intra-species *aggression*, therefore becoming complementary. In this way, the behavioral expressions of a*ggression* have acquired the social function of determining in the adversary an emotional state of fear, and the behavioral manifestations of fear have acquired the social function of reducing the activation of the emotional system of anger in the adversary that threatens, pacifying the contender (Price and Sloman, 1987; Gilbert, 1992).

The complementarity of RAGE and FEAR systems is an important stage of phylogeny, made possible by the evolution of the *limbic system*, of which the *amygdala* is one of the parts (Behrendt, 2011). Species that have this extremely rudimentary structure (*amygdala)*, like the most ancient reptiles, do not have the ability to have social life; therefore, the regulation of access to food and sexual resources takes place through a rudimentary *territorial agonism* (Behrendt, 2012). The characteristic of this competitive system is that the competition between two members of the same species ends with one fleeing from the territory on which the other will dominate (Behrendt, 2012).

The evolution of the *limbic system* allowed the individualized recognition of conspecifics among mammal groups. This evolutionary acquisition allowed the two contenders to share the same territory, without one having to flee, but continuing to cooperate for the common defense from predators and to hunt: *Territorial agonism* has turned into a *ritual agonism*. The behaviors of fear manifested by the contender have become the signal of recognition of the superiority of the adversary, the *submissive behavior*, in order to put an end to hostilities. For this purpose, the FEAR system has coopted behavior from other behavioral and emotional systems, for example, the loser shows his willingness to offer himself as a sexual object to the winner as a *submissive behavior* with which to pacify the adversary (Eibl-Eibesfeldt, 1984). The pacification phase is preceded by a real confrontation phase, in which the two adversaries evaluate each other’s power. This can happen both at a distance, showing your body, inflating it, placing it in poses that enhance its power to the opponent’s eyes, or through a real test of strength at a close range. The fundamental point is that between mammals and birds, in which the ritual agonistic system has evolved, the elimination of the opponent is not foreseen (Lorenz, 1963). The defeated, therefore, manifests his own recognition of the greater power of the adversary and this gives rise to the structuring of what is called *Dominance rank*. The dominance hierarchy implies that high-ranking animals have preferred access to food and mate.

The *Dominance* hierarchy has therefore become the central regulator of the relations between adult individuals in the group of mammals. This structure, to be such and to maintain itself in time, implies that there is a continuous exchange of signs among the members of the group, aimed at maintaining the structural organization of the group. The *submissive behaviors*, described above, thus become part of what are called *yielding subroutines* (Gilbert, 1992; Sloman, 2002) through which the lower-ranking individual constantly signals the recognition of the others superiority. *Yielding subroutines* are based on an active-alert state of high stress characterized by a high concentration of *cortisol*, *low serotonin*, and *testosterone* (Sapolsky, 1983, 1986). Vice versa in the dominant individuals, the presence of *cortisol* is lower, and the concentration of *serotonin* and *testosterone* is higher; therefore, they appear more relaxed but at the same time making losers aware of their dominance through periodic threatening behaviors. These types of behaviors are called *dominance subroutines* (Gilbert, 1992; Allan and Gilbert, 1997).

In the human species, the *Dominant and Submissive motivational/emotional system* is homologous to the one described above. The human emotions homologous to *submissive behaviors* are linked to the impossibility of achieving the goal of domination, that is, Social defeat, which highlights either its own inferiority toward an opponent or the impossibility to achieve a pre-established social purpose (Bjorkqvist, 2001; Rohde, 2001; Allen and Badcock, 2003). The human emotions belonging to the *submissive behaviors* are *fear of judgment, shame, humiliation, sadness from defeat*, and *envy*. The human emotions homologous to dominance behaviors related to the ability to achieve the goal of domination are *anger, triumph, pride, contempt*, and *superiority* (Gardner, 1982; Johnson and Carver, 2012). Evolutionary psychologists and psychiatrists emphasize a further development of the *Agonistic behavior* that is present in primates and especially in the human species. It would consist in the substitution and/or integration of the agonist behavior based on the comparison of the body strength with an agonism based on the ability to create alliances and affiliations, called *hedonic competition* (Chance, 1967; Gilbert, 1992; Price, 1992; Stevens and Price, 1996). The *hedonic competition*, through which the relationships of dominance and rank are structured, is undoubtedly the one most present in human interactions and at the basis of the evaluation of one’s own self-esteem. Individuals with greater relational resources or greater intellectual resources are able to attract the attention of other subjects and then they would obtain a more dominant position in the group. It is to note that progressive corticalization of the human species would have been the effect of pressure to solve the problem of group living and therefore of dominance rank (Humphrey, 1976).

### The Correlation Between Psychoanalytic Aggression and Affective Neuroscience

Freud, even before explicitly defining aggression as one of the components of the *instinct of self-preservation* or *Ego instincts* (Freud, 1910, 1915a), described the instinct of *cruelty* as intimately connected with the instinct of *appropriation.* Freud quotes “The cruel component of the sexual instinct develops in childhood even more independently of the sexual activities that are attached to erotogenic zones. Cruelty in general comes easily to the childish nature, since the obstacle that brings the instinct for mastery to a halt at another person’s pain—namely a capacity for pity—is developed relatively late. The fundamental psychological analysis of this instinct has, as we know, not yet been satisfactorily achieved. It may be assumed that the impulse of cruelty arises from the instinct for mastery and appears at a period of sexual life at which the genitals have not yet taken over their later role” (Freud, 1905, p. 193). Freud describes *cruelty* as an instinct that arises in childhood, from the instinct for *Mastery* (*Bemächtigungstrieb*), that is, from the desire of the subject to take possession and dominate the object, that being the other person. During the first phase of ontogenetic development, *pity* has not yet developed, without which, the sufferance of others cannot be perceived, therefore, cruelty prevails and like in *territorial agonism*, the dominant opponent cannot recognize the submission signals of the defeated contender and so is unable to restrain himself.

In Freud’s exposition on *cruelty* (Freud, 1905, p. 193), we can find aspects of the motivational/emotional RAGE system described above, namely the *inter-mail aggression* (*Dominance/Submission motivational/emotional system*) in its primordial form of *territorial agonism*, where the motivational drive for the complete *mastery* of a territory is in force. Another component of the RAGE system is also visible in the instinct of *mastery* and *cruelty*, namely *predatory aggression*. *Predatory aggression* is an appetitive behavior for exploring and mastering prey. *Predatory aggression* springs from the same source of SEEKING behavior (dopaminergic system) and both are subjectively pleasurable and elicit high levels of self-stimulation (Panksepp and Zellner, 2004). Freud again in *The Three Essays on Sexual Theory* puts *impulse of cruelty* to the *instinct of scopophilia* side by side. Freud quotes “sexual development and the development of the instinct of scopophilia and cruelty are subject to mutual influences…” (Freud, 1905, p. 193 footnote 1). Freud seems to suggest that *instinct of scopophilia* (pleasure from looking) is intimately connected to the *impulse of cruelty* and then to the instinct of *mastery*. The SEEKING system has the tools in the sense organs through which the exploratory drive can take place. In the human species, the sight has undoubtedly replaced the importance that the smell has for most of the mammals (Hughes et al., 2014) both in seeking and in the sexuality. But in all the mammals looking and gazing are also means of competitive engagement (Chance, 1967, Baron-Choen, 1995) but also the vehicle to display *submissive behavior* avoiding direct eye contact with the dominant contender (Eibl-Eibesfeldt, 1984). The sense of sight is connected with *envy* (from Latin *invidia*, from *invidere* “regard maliciously, grudge,” and from *in-* “into” and *videre* “to see”), which expresses the maliciously intention of taking possession of what a conspecific possesses, as Klein masterly described in the psychoanalytic field *Envy and gratitude* (Klein, 1957). *Envy* is an emotional expression of the subject frustrated in his desire to possess a matter owned by another subject, that is, “the aspirations of the SEEKING system are thwarted…” (Panksepp and Biven, 2012, p. 149); see also the equivalence between SEEKING system and Freud concept of *libido* in Solms and Turnbull, 2002). *SEEKING* system frustrated arouses the *affective attack*, the third component of the RAGE system (see above). The mind of a child or of a psychotic is particularly vulnerable to *envy* in the presence of those who own and enjoy desirable riches (for example, material goods but also intelligence, friendship, etc.). And so *envy* becomes the source of a devastating rage that wants to annihilate those who hold such resources and in turn causes the individual to become paranoid, expecting to receive similar annihilating attacks. This is the mental state described by Klein as a schizo-paranoid position (Klein, 1957). The substitution of the concept of *stage* with *position*, which implies the possibility throughout the mental life of possible oscillations between the two *schizo-paranoid* and *depressive* positions (Bion, 1963), is undoubtedly one of Klein’s most interesting conceptualizations. The *schizo-paranoid* versus *depressive positions* are two ways in which the subject’s psyche manages the problem of *aggression* (see above) and which can be compared to the oscillations between two types of *social competition*. The *schizo-paranoid position* can be seen as an expression of the archaic, territorial Agonism, in which rage prevails characterized by an aggression aimed at making the other contender disappear from his own sight, from his own “territory,” in which there is no limitation to the desire of *mastery* and *cruelty*. The *depressive position* can be seen as an expression of the second type of social competition aimed at the formation of *Dominance hierarchy*, in which the drive to *mastery*, *cruelty*, and *dominance* without limits is inhibited from the *submissive behaviors* of the adversary defeated. This modulates and restrains the winner’s intention to annihilate and send away from his own sight the opponent forming a *Dominance hierarchy* in which both the winner and the defeated cohabit together, and so making it possible for the two contenders to stay close on the same territory and to cooperate together, living in the same group (see the *motivation to cooperate* in Tomasello and Gonzalez-Cabrera, 2017).

Freud sought to find the roots of aggression almost since the beginning of his construction of psychoanalytic theory, giving rise to observations and theoretical elaborations that have then been resumed and amplified by his successors. Above we have considered the work of Klein, now we want to consider the work of Hans Kohut (1913–1981), who took up the theory of aggression that what mentioned in Freud’s theory of Narcissism.

In Freud’s (1914) *Introduction to the Narcissism*, he traces the primordial roots of aggression in hate that is stimulated in the subject by the perception of objects external to the narcissistic ego and the reaction of displeasure caused by them (see above pg 5). The hate so considered belongs to the *self-preservation instincts* or *Ego instincts*, “Hate… always remains in an intimate relation with the self-preservation instinct” (Freud, 1914, p. 139). Kohut studied the narcissistic ego reaction to external stimuli and reached the conclusion that *hate* and the *aggression* are effects of the psychological defense against the *narcissistic wound* and *narcissistic blow* and not connected to an instinctual functioning. Kohut quotes “In essence then, I believe that man’s destructivenss as a psychological phenomenon is secondary… Aggression, furthermore, as a psychological phenomenon, is not elemental. Like the inorganic building blocks of the organic molecule, it is, from the beginning, a constituent of the child’s assertiveness, and under normal circumstances it remains alloyed to the assertiveness of the adult’s mature self” (Kohut, 1977, p. 116). The roots of aggression are in the insufficiently empathic relationship among caregivers and child: “destructiveness… arises originally as a result of the failure of the self-object environment to meet the child’s need for optimal… emphatic responses” (Kohut, 1977, p. 116). As a consequence, the subject who has suffered a *narcissistic wound* is vulnerable to social competition in which, instead showing an assertive aggression, he will exhibit a *Chronic Narcissistic Rage* (Kohut, 1972). So the *Chronic Narcissistic Rage* derives from a CARE system (Panksepp, 1998) not sufficiently adapted to the child’s *Attachment* needs, determining a chronically activation of SADNESS system (Panksepp, 1998), which triggers the *defensive attack* of RAGE system (Panksepp, 1998). The social competition is experienced as fear of deadly oppression with acute sense of inferiority, which actives *shame and narcissistic rage* (Kohut, 1972). The activation of two latter prevents the subject from recognizing his own real defeat, and therefore the possibility of implementing a submission/pacification behavior that is functional to stop the attack of the antagonist dominant, with the consequent attack by the latter in a continuous vicious cycle. Hence, the narcissistic and borderline personality disorders, but also the manic mood disorders (see below).

We have considered how both Freud and his successors place the roots of aggression in childhood. The aggression in childhood has also been confirmed through research carried out in PLAY system (MacLean, 1985; Panksepp, 1998; Panksepp and Biven, 2012). In the ontogeny of mammals, PLAY system has the function of training the individual to regulate social and competitive interaction with the other conspecifics (Panksepp, 2008). MacLean highlights that the play has the function “to promote harmony in the nest” (MacLean, 1985), and also Panksepp highlights how PLAY system evokes positive feelings, despite being a form of training for dominance/submissive interactions (Panksepp et al., 1985; Panksepp, 2008). PLAY system is characterized by role reversals, that is, the winning contender does not remain the dominance figure indefinitely, and allows the weaker defeated antagonist to exchange roles periodically (Panksepp and Biven, 2012). With sexual maturity and testosterone production, this role reversal tends to decrease within competitive interactions and at the same time, testosterone causes the individual to become more suspicious toward his/her conspecifics (Siegel, 2005). In adolescence, as we will see below, the tendency toward peer play becomes more of an explicit social competition to gain dominance. Physical contact is one of the motivations for both animal and human species during the play (P-Biven), highlighting the fact that play is closely connected to the attachment system. PLAY system trains young offspring for social competitions in which they learn to remain included in relationships so as to learn social rules, also preparing them from an early age (Mascaro and Csibra, 2012) to face social competition (Panksepp et al., 1985; Hawley, 1999; Hawley, 2007).

### Aggression, Fear, and Psychopathology: From Attachment to Dominance

The theory of motivational/emotional systems, based on a phylogenetic view of the ontogenetic development of the human brain, implies, therefore, that the human species shares with the other mammals the innate systems for regulating inter-individual relations.

The GRIEF/SADNESS and CARE motivational/emotional systems (Bowlby, 1969; Panksepp, 1998) are those that regulate the relationship between the new born and the caregivers, but they are also those that allow an adult to ask help from other adults. Furthermore, in human species, the GRIEF/SADNESS and CARE motivational/emotional systems become integrated with the LUST system to create the *romantic love* (Hazan and Shaver, 1987; Simpson, 1990) and so the lasting couples for the care of the offspring (Eibl-Eibesfeldt, 1984). Finally, there are *Dominance and Submissive motivational/emotional system*, which has been widely discussed.

The attachment system (or GRIEF/SADNESS in the Panksepp theory, 1998) has become in recent decades, as mentioned in the introduction, one of the main factors in which to look for the etiopathogenesis of mental suffering. Bowlby’s work traced the lines of this “belief” (Cowan and Cowan, 2007), while the research data in this field have gradually highlighted the weakness of the attachment construct (Cowan and Cowan, 2007).

Bowlby and subsequent researchers have shown that attachment is not a simple response to external stimuli but a well-organized behavioral system (see, e.g., Hofer, 2014), so in conditions of threat a puppy as well as a child develops a stable strategy with the same objective: seeking comfort toward an adult who can function as a *secure base*. This *secure base* would be a factor of resilience toward subsequent stressors or traumas (Sroufe and Siegel, 2011). The research on attachment has thus highlighted the instinctual basis of this behavioral system on which the good functioning of the personality is based, and the investigation on interactions between genes, social environmental factors, and individual history has now shown the overcoming of the old counterpoint between *nature vs. nurture* (Suomi, 2000). At the same time, research has begun to show the weakness of the assumption that the attachment developed in childhood was only connected to one caregiver, that is the mother, but instead there is a specific *attachment working model* for each caregiver with which the child has had significant relations (Fox et al., 1991; Sagi et al., 1995; Asendorpf and Wilpers, 2000). Instead it seems to be more sustainable that there are more *attachment working models* that have developed for different attachment figures in a continuous dimension of security rather than categorial ones (Alexandrov et al., 2005). Equally, weak is the way in which attachment remains stable as a working model for life (Grossmann et al., 2002; Zhang and Labouvie-Vief, 2004; Sroufe, 2005). Finally, notwithstanding Bowlby (1979) recognized that the attachment system undergoes a modification during development, from parental regulation through dyadic regulation to self-regulation. This developmental dynamic has been little studied and in particular in the transition from childhood to adolescence and adulthood, the scholars have preferred to take the attachment system with an unchanging “package” in time once formed (Cowan and Cowan, 2007).

The attachment system has thus remained an isolated etiopathogenetic factor in the time of development, not considered in the interaction with sexual growth. On the contrary, sexual maturity determines an intense readjustment in the dynamics of motivational/emotional systems that regulate relationships. Sexual development leads to maturity, as indicated above, *Dominance and Submissive motivational system* that is completely different from attachment and more phylogenetically archaic than that, with a specific relational “grammar.” Neurobiology and developmental endocrinology indicate that puberty gives rise to a real new course both in the functioning of the organism of young individuals and in their behaviors (Tottenham and Galván, 2016).

In every species of mammals, the onset of puberty (Sisk and Foster, 2004; Schneider, 2013) causes behavioral changes by the maturation of the sexual organs in individuals. Studies in animals have now amply demonstrated the correlation between the levels of androgenic hormones and the levels of *aggression* in males (Beeman, 1947; Simpson, 2001). In the human species, the influence of the puberty development and androgen hormones on aggressive behavior is undoubtedly much more complex to study. Meta-analysis studies on research-related works on the link between hormones and *aggression* have shown a positive correlation between levels of the androgens and the aggressiveness present in male subjects (Archer, 1994; Book et al., 2001). An important contribution was given by Mazur and Booth (1998) who, revising a vast literature, introduced the distinction between aggression and dominance in men. Testosterone levels correlate positively with the dynamics of dominance, of which aggression is only an expression. Research on animals has shown that even in females*, aggression* correlates with the levels of the androgens (Van de Poll et al., 1988) and also in the human species (Dabbs et al., 1988).

Puberty and sexual maturation determine changes in neurophysiology and therefore in adaptive behaviors, by a complex interaction between gonadal hormones, dopaminergic system, and stress axis (Sinclair et al., 2014). In puberty, the maturation of androgen hormones increases dopamine synthesis and stimulates social *aggression* (Schulz et al., 2006; Wahlstrom et al., 2010). At the same time, the androgen hormones promote the increase of number glucocorticoid receptors in dopaminergic system, both in prefrontal cortex PFC, in hippocampus and in striatum (Schulz et al., 2006; Sinclair et al., 2014). These data give reason for behavioral changes in male adolescence. Testosterone also increases the sensitivity of the amygdala to social threatening stimuli (Derntl et al., 2009; Bos et al., 2012). So, in adolescence, there is an increased sensitivity of the FEAR system toward social stimuli. In the adolescence, the positive correlation among gonadal maturation, the increase of testosterone production, and the pushing to dominance behavior on the one hand and on the other an increased sensitivity of the FEAR motivational system for social stressors (Steinberg, 2007; Steinberg, 2008) led to the discovery of the *ratio* between testosterone and cortisol (Terburg et al., 2009; Van der Westhuizen and Solms, 2015) that correlates significantly with the dominance behavior.

Summarizing, first, the theory of social competition, based on *Dominance and Submissive motivational/emotional system*, circumscribes and qualifies a theory of *aggression* that contains within it, in a complementary and functional way, an equally circumscribed, and qualified theory of social fear also named Social defeat (Hollis and Kabbaj, 2014). In animal models, *Social defeat* generates persistent emotional stress without habituation (Tidey and Miczek, 1997; Moxon, 2009; Hollis and Kabbaj, 2014). The experience of *Social defeat* causes tachycardia and hyperthermia, elevated blood pressure, increased adrenocorticotropic hormone (ACTH), and corticosterone levels (Tornatzky and Miczek, 1993). The repeated exposure to *Social defeat* caused decreased locomotor and exploratory activity, reduced *aggression* and reduced sexual behavior, and increased *submissive behavior* and anxiety (Meerlo et al., 1996; Tidey and Miczek, 1997; Ruis et al., 1999; Crawford et al., 2013; Hollis and Kabbaj, 2014).

Second, the *Dominance and Submissive motivational system* is expressed through innate emotional models, which in subjects determine the activation of complementary and automatic responses.

Finally, *Dominance and Submissive motivational system* has its particular activation in the development stage of puberty, functionally connected to the sexual hormones and brain maturation.

All the peculiarities of the *Dominance and Submissive motivational system* in animal models can be found in *homologous* way in the human species, both in the “physiological” psycho-social dynamic of social competition and in their extreme forms in psychopathology (Johnson et al., 2012).

As mentioned above, the interactions related to social competition are present from the first year of life (Sheridan and Williams, 2006), but before puberty, the emotional homeostasis disrupted by Social defeat was continually reestablished by the *Attachment motivational system*, which allowed the child to find a *safe haven* again for relational perturbations in the bond with caregiver. Instead, the adolescent perceives the impossibility to experiment the previous solution to manage the *Social defeat* to which the sexual maturation of the *Dominance and Submissive motivational systems* exposes him or her. This could explain one of the main reasons for which the psychopathology appears and stabilizes in the adolescence stage.

It is within this new *biological value systems* (consequence of pubertal sexual maturation, Edelman, 1992) located in the *brain stem, basal ganglia*, *and limbic system* (MacLean, 1990a,b; Panksepp, 1998; Edelman et al., 2011) that the adolescent can begin to perceive *nameless dread* (Bion, 1962a). The adolescent can begin to evolve an increasingly strong conviction to be unable to gain a place within the human group and to form and maintain emotional ties, that now are mediated by his capacity to obtain a position in the rank hierarchies (school, peers, sport, etc.) in which the group is organized outside the primary family group. It is at this point in life that anguish can become *nameless dread* (Bion, 1962a) or *catastrophic dread* (Bion, 1970) because of the massive and automatic subcortical activation of emotions connected to the expression of the *Dominance and Submissive motivational system* making the subject *spectator* of his/her own states of relational fear or impulsivity and euphoric state that until a short time before, in infancy, had not been experienced. When social interactions activate the *Agonistic behavior*, the subject can perceive himself as “destined to victory” or “destined to defeat” (Sloman, 2002). The developmental history of the subject determined by an inseparable mix of genetic endowments and experiences connected to the *Dominant and Submissive system* since the first year of life will determine how in adolescence and then in adult life the subject will react to the stressors of social competition. If the activation of the emotions connected to the *Agonistic behavior* exceed the subjective tolerance threshold, giving rise to excessive stress, activate either behaviors connected *to* the involuntary defeat strategy or involuntary dominant strategy (Price, 2002; Sloman, 2002). The first is the backdrop of depression. The depressed subject feels himself a failure and loser (Gilbert, 1992), whereas a manic person would feel successful and victorious (Johnson and Carver, 2012), without recognizing his/her true mental state unsuitable for facing the real situation and therefore self-inflicting relational frustrations and therefore mental pain. The borderline personality disorder is a combination of the two involuntary strategic (Kupferberg et al., 2016; Stone, 2013). A person with borderline personality disorder shows an extreme susceptibility to social competition, feels easily criticized, scared of failure and shame, with the simultaneous activation of both the involuntary defeat strategy and involuntary dominant strategy, switching rapidly from deescalation (depressed position) to escalation (aggressive and manic behavior), and vice versa (Williams, 2017).

## Conclusion

Psychoanalysis has, in its theoretical essence, the fundamentals of evolutionary thought, which have been almost completely covered and hidden by the *psychological drift* that has prevailed in the psychoanalytic field. For this, following the indications of Bowlby (1969), it seems very useful for psychoanalysis to return to focus on the instincts or motivational/emotional systems that organize the human mental and relational life, since their study can give psychodynamic research that scientific foundation which is currently one of the most its onerous problems. The motivational systems through which relationships are organized in childhood and later in adolescence and adulthood, are primarily the GRIEF/SADNESS and CARE systems and then, with maturation of LUST system, the *Dominance* and *Submissive system*, with very peculiar emotional characteristics and manifestations. The *Agonistic behavior* has the characteristics, as described above, of covering most of the human phenomenology connected to the concept of *aggression*. The aggressiveness of the *Dominance* and *Submissive motivational system* is strongly “ritualized,” closely and structurally connected to the complementary ritualized fear emotional behaviors.

Lorenz (1963) states that the process of individualization, or the ability of an individual to recognize one’s own conspecific, has evolved as a function of intraspecious aggressiveness, which has successfully fulfilled three central functions for the survival of the species: first, to distribute the individuals of the same species on a territory in a functional way to the resources (territorial aggressiveness), second, to select the most suitable individuals for the perpetuation of the species (sexual selection), and finally, to favor the care and defense of the offspring (Lorenz, 1963). There are, however, also animal species in which there is no intraspecious *aggression.* These species have a gregarious instinct, which allows more individuals to keep close to each other, like the large shoal of fish, which Lorenz calls “anonymous ranks,” without there being the ability to recognize the neighbor and distinguish him from another. This capacity would have started, however, starting from the territorial aggressiveness, connected functionally to the identification of the adversary as belonging to the same species and to the same genus and therefore the ability to recognize the various aspects related to the evaluation of the competitive resources of the other. The evolution, then, of the *territorial agonism* in *agonism for the dominance of rank* among more individuals copresent in the same territory, has been functionally connected to a more continuous recognition of the other, identified in the dominant or submissive relational position within the group to which they belong. In the course of phylogeny, therefore, the ability to recognize one’s own conspecific has been increasingly selected in order to establish a correct distance between them.

The recognition of the same species activates in the conspecific agonistic *aggression* (Lorenz, 1963), and this happens because the individual makes himself clearly visible in the eyes of the other. Regarding the previous sentence, Lorenz’s descriptions of animals that are defeated in the competitive area, quickly fade their colors before which they were extremely intense. That which has been described by the great ethologist could be more than a suggestive metaphor, usable to understand both the emotional state and the care of the suffering human mind. The evolutionary and developmental processes called individuation or subjectivation (De Monticelli, 2008), see the subject engaged in the hard struggle to gain visibility and recognition within the group to which he belongs. This is often related to a state of deep fear, which makes this personal developmental process extremely painful or impossible and therefore could provoke anxiety, mood, and personality disorders. The conceptualization of *aggression* as *intraspecies aggression* could give coherence to mental states, behaviors, and emotions that characterize the various nosographic pictures and can constitute a theoretical-clinical model of great heuristic force.

Even during childhood, hierarchies of peer dominance are formed (Havely, 2007), in which children can feel the emotions connected to being dominant or submissive. But, as considered above, at this age, the immaturity of the sexual system and the priority of the attachment system in the hierarchy of motivational/emotional systems mitigate the presence of emotions connected to the *Dominance* and *Submissive motivational system*. PLAY system has a central position in the development of *Dominance* and *Submissive motivational system* and, in connection with the attachment system, mitigate the emotions of social competition among peers, making it possible to exchange the positions of winner and loser during the competitive interactions.

As seen in this article, the link between childhood and aggression has been a key factor throughout the history of psychoanalysis. The asymmetric relationship between children and their parents exposes the former to the danger of being annihilated by the action of latter causing the aggressive emotions in the offspring, as explained initially in Freud’s theories concerning Oedipus and castration complex. Authors such as Hans Kohut (Kohut, 1972), Donald Winnicott, or Wilfred Bion (each one having had different opinions) stated that the nonempathic relationship (Winnicott, 1960; Kohut, 1972) or insufficient *Reverie* (Bion, 1962b) with parents was the cause of offspring vulnerability toward self-control of aggression in social competition. Psychoanalyst John Bowlby associated this vulnerability with the failure of the instinctual CARE system thus impeding a *secure* attachment (see, e.g., Bowlby, 1944). So, psychoanalysts could have been misled by the theory that the roots of pathogenic aggression took hold during childhood. The problem of aggression/fear according to the model of *Dominance and Submissive motivational/emotional system*, that has its specific activation in adolescence, first of all indicates the need to reposition the etiopathogenic conflict from the inter-family dimension to the relationship subject-world outside the family. This allows, secondarily, a greater contextualization of the genesis of mental illness, being able to identify which environmental aspects “collude” etiopathogenetically (Gilbert et al., 2007) with the change in the subject from the primacy of the attachment motivational system to the agonistic one. Finally, the model presented here could indicate the need to reconsider some points of the clinical theory in psychoanalysis, for example, that of *transference* or that of *insight,* for which some psychoanalysts have already been engaged for some time (see, e.g., Gabbard and Westen, 2003; Pally, 2007; Allen et al., 2008), indicating ways that are more compatible with epistemology as proposed in this work.

## Author Contributions

TG is the main author—development of hypotheses and concepts. US is the coauthor—critical discussion of literature and concepts.

### Conflict of Interest Statement

The authors declare that the research was conducted in the absence of any commercial or financial relationships that could be construed as a potential conflict of interest.
